# Performance-optimized otoplasty

**DOI:** 10.1186/s12893-022-01587-y

**Published:** 2022-05-14

**Authors:** Marcelo Souza Assis, Leila Souto Miranda

**Affiliations:** Orelhinha Institute, 55 Oriente ST, Chácara da Barra, Campinas, São Paulo, 13090-740 Brazil

**Keywords:** Ear correction, Otoplasty, Outpatient surgery, Plastic surgery

## Abstract

**Objectives:**

This study proposes a new surgical alternative for the most common deformity in the ears, the so-called "protruding/prominent ears", which is a condition that affects 5% of the Caucasian population (Goulart et al. in Rev Bras Cir Plast 26:602–607, 2011). This technique comes with the benefits of reduced surgical time, shallow learning curve, and a low revision rate.

**Methods:**

We studied a total of 213 patients with an indication for otoplasty from January 2020 to January 2021. Women made up 65% of the study population, while men made up 35%, with an average age of 21 years, the youngest being 7 years of age. The technique presented here corrects all the deformities that cause protruding ears and can be performed together with other ear surgeries, such as surgical treatment of macrotia and lobuloplasty. All surgeries were performed in an outpatient setting under local anesthesia and sedation.

**Results:**

All surgeries followed a performance-optimized protocol, with an average total surgical time of 45 min for a bilateral approach. Revision surgery was needed in 2% of cases, with the most frequent complaint being asymmetry in the upper third of the ears. The complication rate was approximately 7.5%, with 1 case of hematoma, 1 case of mild infection, 2 cases of altered ear sensitivity, 3 cases of keloid scar formation, 6 cases of asymmetry in the upper third of the ears, and 3 cases of irregularities or spikes in the antihelix cartilage. Patient satisfaction was measured using the McDowell/Wright Objectives and Outcome Index (McDowell in Plast Reconstr Surg 41:17–27).

**Conclusion:**

The proposed performance technique is a viable alternative to optimize the surgical time of otoplasty in an outpatient setting. This technique can be performed together with other corrective ear surgeries, has a shallow learning curve, and has a low revision rate.

*Level IV:* Evidence obtained from multiple time series with or without the intervention, such as case studies.

## Introduction

Prominent ears result from changes in the cartilage and are often bilateral. This is usually due to genetic and hereditary factors but can also be caused by the fetal position [1]. Prominent ears should not cause bullying but increased public exposure on social media leads to judgment and psychological and traumatic damage [2, 3]. Prominent ears are one of the main causes of bullying due to their physical appearance [3–5]. From Dieffenbach in 1845 to Fritsch in 1992, various corrective ear surgery techniques have been developed [6–9]. Each technique has advantages and disadvantages, such as the techniques using Mustardé sutures to redesign the antihelix, developed in 1962 [10, 11], and the techniques using Furnas sutures for conchal-scaphal fixation, as described in 1959 [8]. This study presents a new option for the otoplasty technique, with an improved surgical time, low revision and high satisfaction rates. Furthermore, the technique allows designing the antihelix through parallel cartilage islands without the need for fixation points with nonabsorbable threads, which, in some cases, lead to suture extrusions [12]. It also requires no major detachments in the mastoid and fixation with Furnas sutures, which can generate chronic pain or local sensitivity changes [8].

## Patients and methods

A total of 213 patients were operated on from January 2020 to January 2021, 136 women and 77 men, all with bilateral intervention, and a mean age of 21 years. Patients were evaluated based on complaints of prominent ears and had an indication for surgery to correct antihelix erasure, conchal hypertrophy or lobe projection, with one or more of these corrections being performed.

The patients were operated on an outpatient basis, without admission to the hospital, and stayed in the hospital for approximately two hours.

All surgeries were performed under local anesthesia using 20 ml saline solution, 5 ml 2% xylocaine, 3 ml 0.5% Marcaine, 0.5 ml adrenaline, and sedation with 0.05 mg/kg midazolam, 2–3 mcg/kg fentanyl, and 10–20 mcg/kg/min propofol. In anxious patients, 1 mcg/kg clonidine was administered, and in children, 0.5–1 mg/kg ketamine was administered if necessary.

**Fig. 1:**
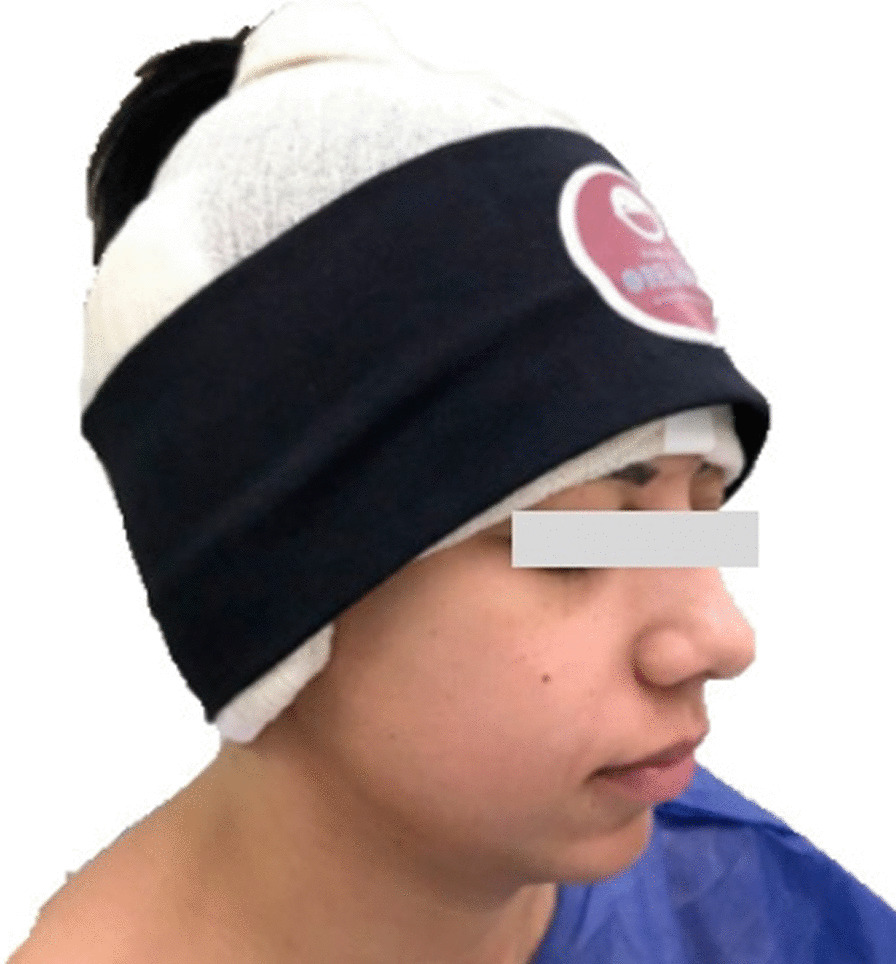
Standardized dressing by the service. Source: personal archive

Cotton molds soaked in saline solution were applied as dressings in the region of the scapha and auricular concha, as well as cotton pads under the ears, and a 10 cm wide orthopedic tubular mesh was used to stabilize the dressing without going through the neck, which was kept for 5 days (Fig. 1).

### Surgical technique

#### 1st Step—marking the ears

**Fig. 2:**
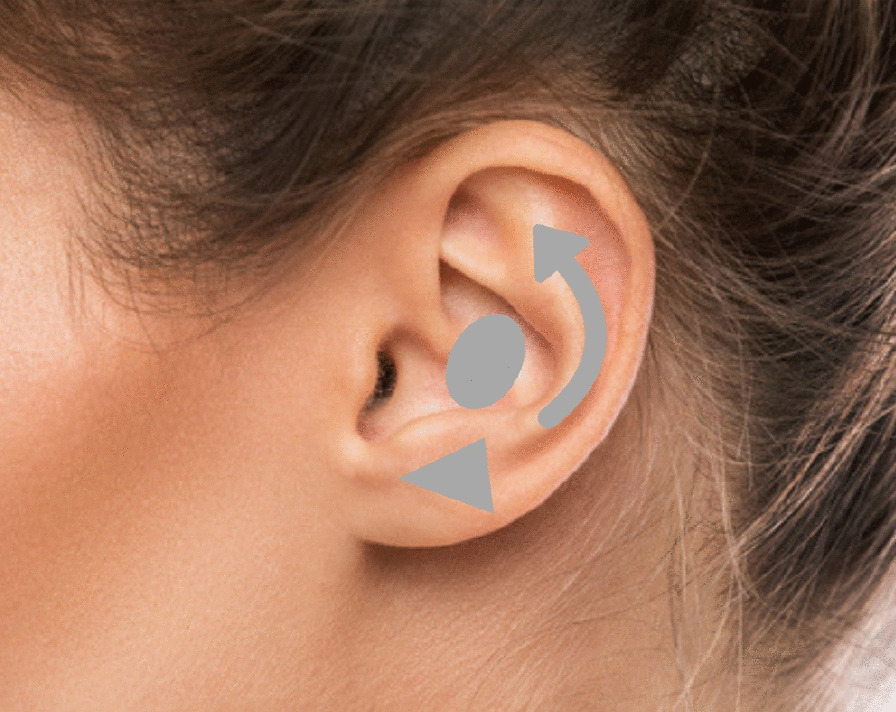
Markings prior to the otoplasty. Source: Shutterstock image license

The patient is marked while sitting and supine. We performed a bidigital maneuver on the antihelix to check its new projection. A retroauricular skin flap is marked, observing the line projection of the antihelix and the inferior border of the auricular sulcus. Conchal hypertrophy is marked during the perioperative period (Fig. 2).

### 2nd Step—infiltration with local anesthetic

After sedation, we performed local anesthesia only in the retroauricular flap marked, with associated skin detachment.

### 3rd Step—surgical procedure

**Fig. 3:**
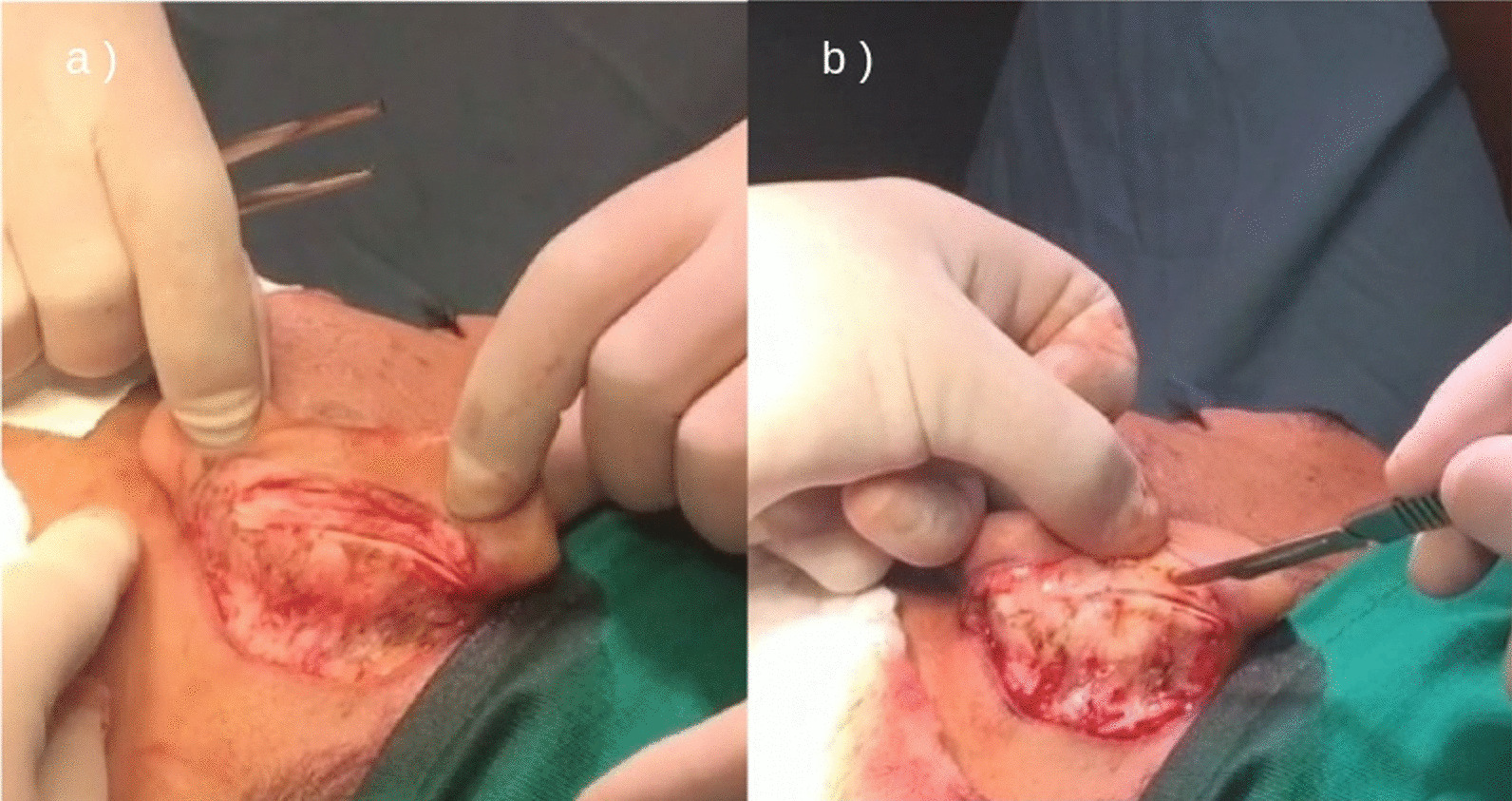
Intraoperative view: **a** Parallel chondrotomy on islands, **b** Preparation of equidistant parallel cartilages of 2 mm for antihelix reconstruction. Source: personal archive

The performance-optimized otoplasty technique consists of three stages of cartilage treatment depending on the needs of each patient and in accordance with the medical indication. We performed 2–3 mm thick, half-moon shaped, parallel chondrotomies on cartilage islands in the antihelix cartilage, following the surgical posterior edge of the ears, with a total depth to the dermis. The objective of this maneuver is to mold the antihelix area into an inverted U-shape, with the first island being the lateral wall, the second, the top; and the third, the medial wall, thus forming a new design by repositioning the upper third of the ears close to the head (Fig. 3).

### 4th Step—conchal cartilage treatment

**Fig. 4:**
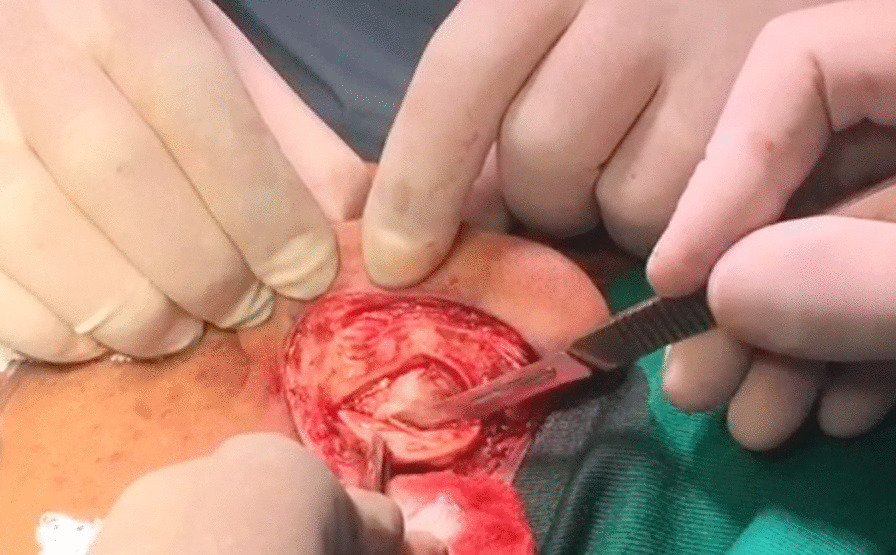
Removal of the juxta-perichondrial auricular concha according to the marking performed. Source: personal archive

After anesthetic reinforcement in the greater auricular nerve and anterior infiltration of the concha for skin detachment, we performed an incision in the conchal cartilage flap to remove its juxta-perichondrial excess (Fig. 4).

### 5th Ste—treatment of the helix

**Fig. 5:**
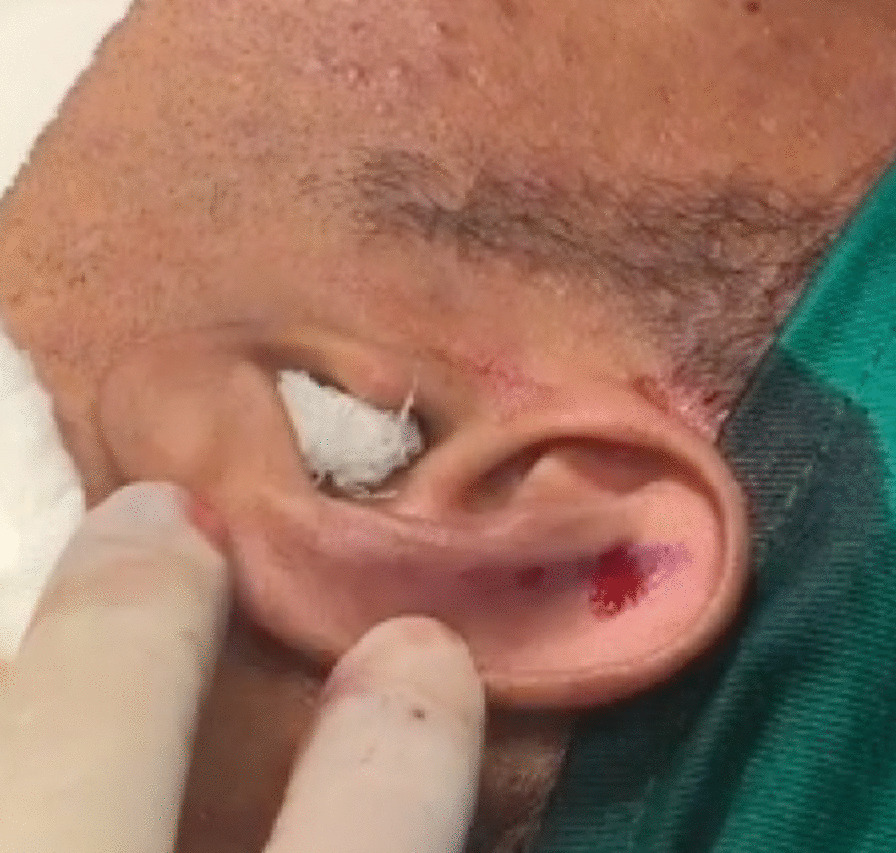
Surgery completed with antihelix reconstruction, auricular concha in position and helix parallel to the middle and upper third. Source: personal archive

When a patient shows an anterior projection of the earlobe, we break the cartilage spring at the antihelix–helix junction, and when indicated, remove the posterior skin excess on the fishtail or combine it with lobuloplasty (Fig. 5).

### 6th Step—skin closure

**Fig. 6:**
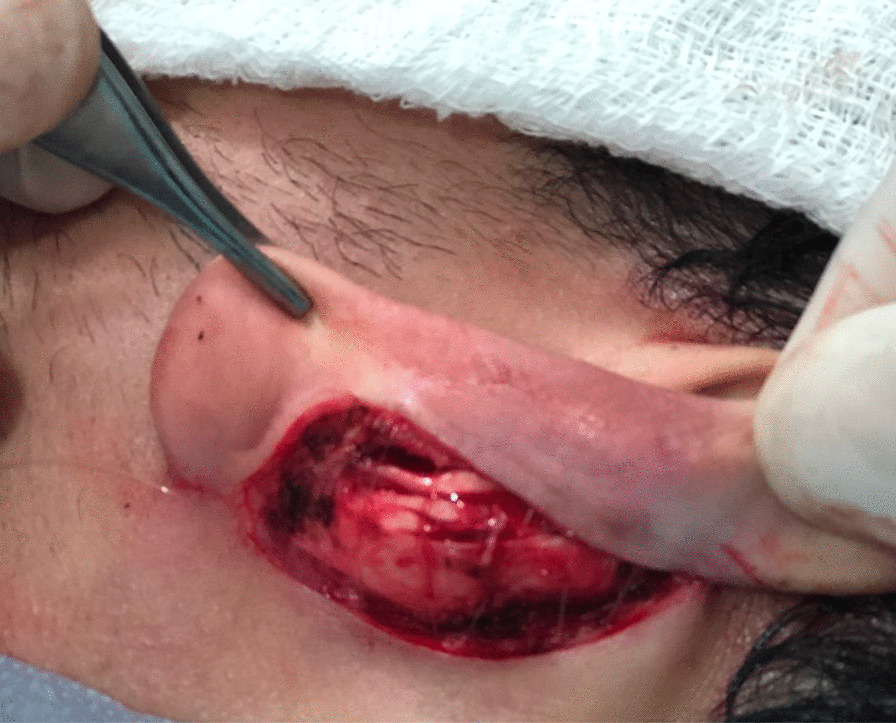
Continuous intradermal suture with absorbable thread. Source: personal archive

After properly reviewing hemostasis, we performed a reinforcement stitch in the upper third of the posterior region of the ears, 1.5 cm from the open upper margin, between the area above the chondrotomies and the mastoid, with a 4.0 monocryl suture. This procedure had the purpose of reducing the recurrence of spikes in the upper third, which is the main cause of asymmetry, and stabilization of cartilage islands on the antihelix. The closure is performed with monocryl 4.0 suture in continuous intradermal suture (Fig. 6).

### 7th Step—cotton mold and bandage helmet

**Fig. 7:**
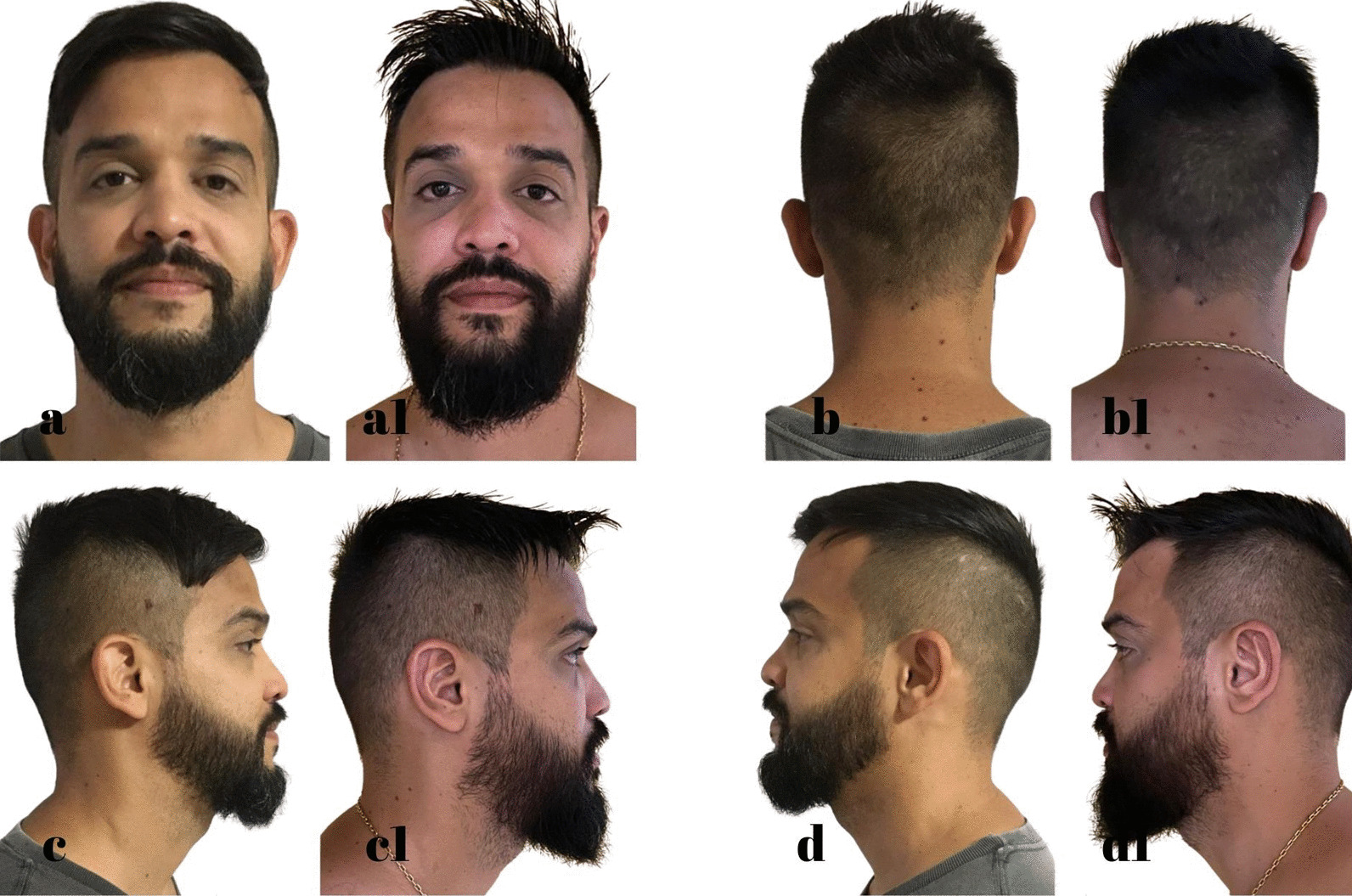
S.G.S. 29 years, **a**, **b**, **c**, **d** Before otoplasty. **a1**, **b1**, **c1**, **d1** 6 months after surgery. Source: personal archive

**Fig. 8:**
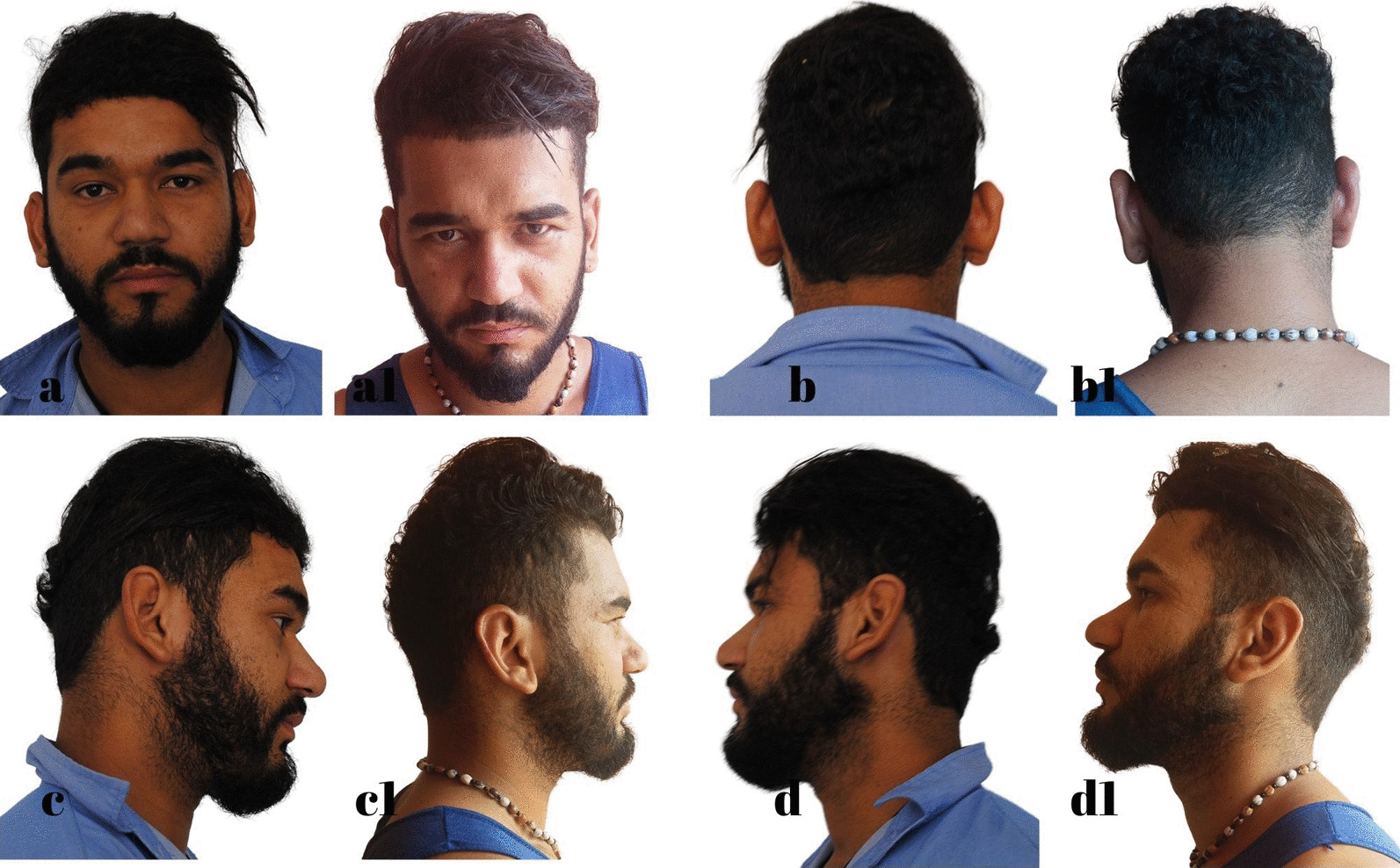
L.R.S. 32 years, **a**, **b**, **c**, **d** Before otoplasty. **a1**, **b1**, **c1**, **d1** 1 year after surgery. Source: Personal archive

**Fig. 9:**
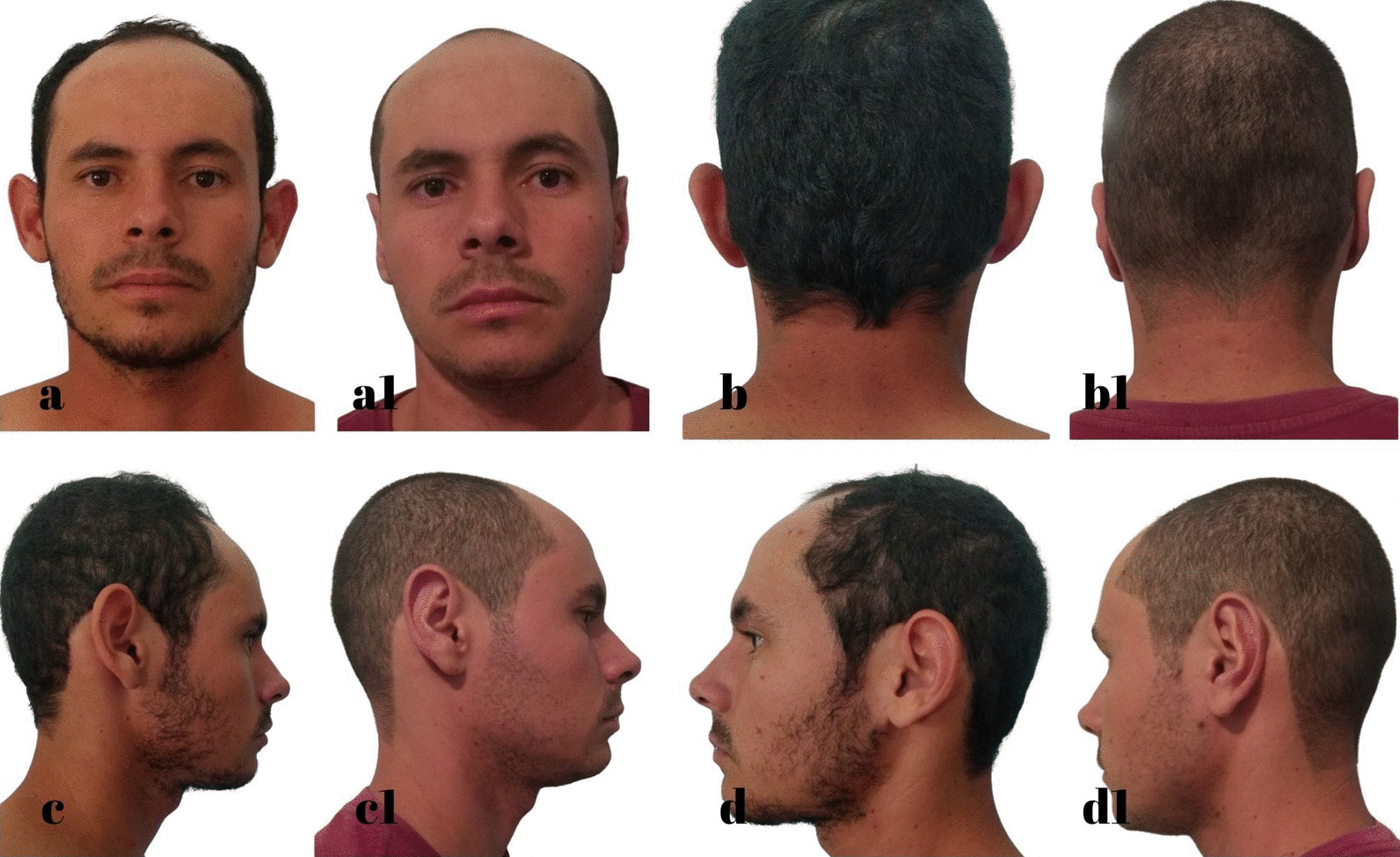
A.O.C. 36 years, **a**, **b**, **c**, **d** Before otoplasty. **a1**, **b1**, **c1**, **d1** 11 months after surgery. Source: Personal archive

**Fig. 10:**
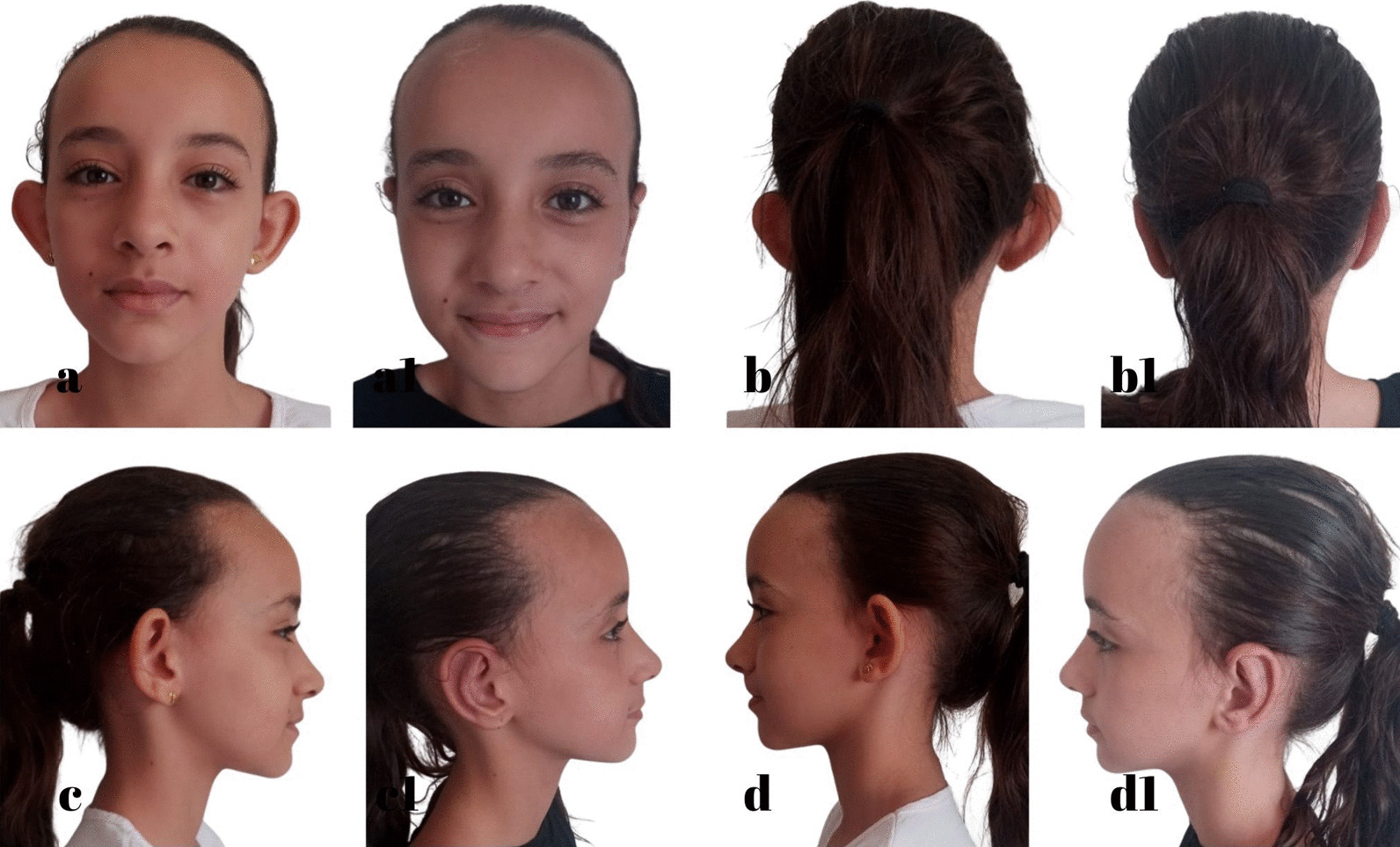
I.A.F. 9 years, **a**, **b**, **c**, **d** Before otoplasty. **a1**, **b1**, **c1**, **d1** 1 year after surgery. Source: Personal archive

**Fig. 11:**
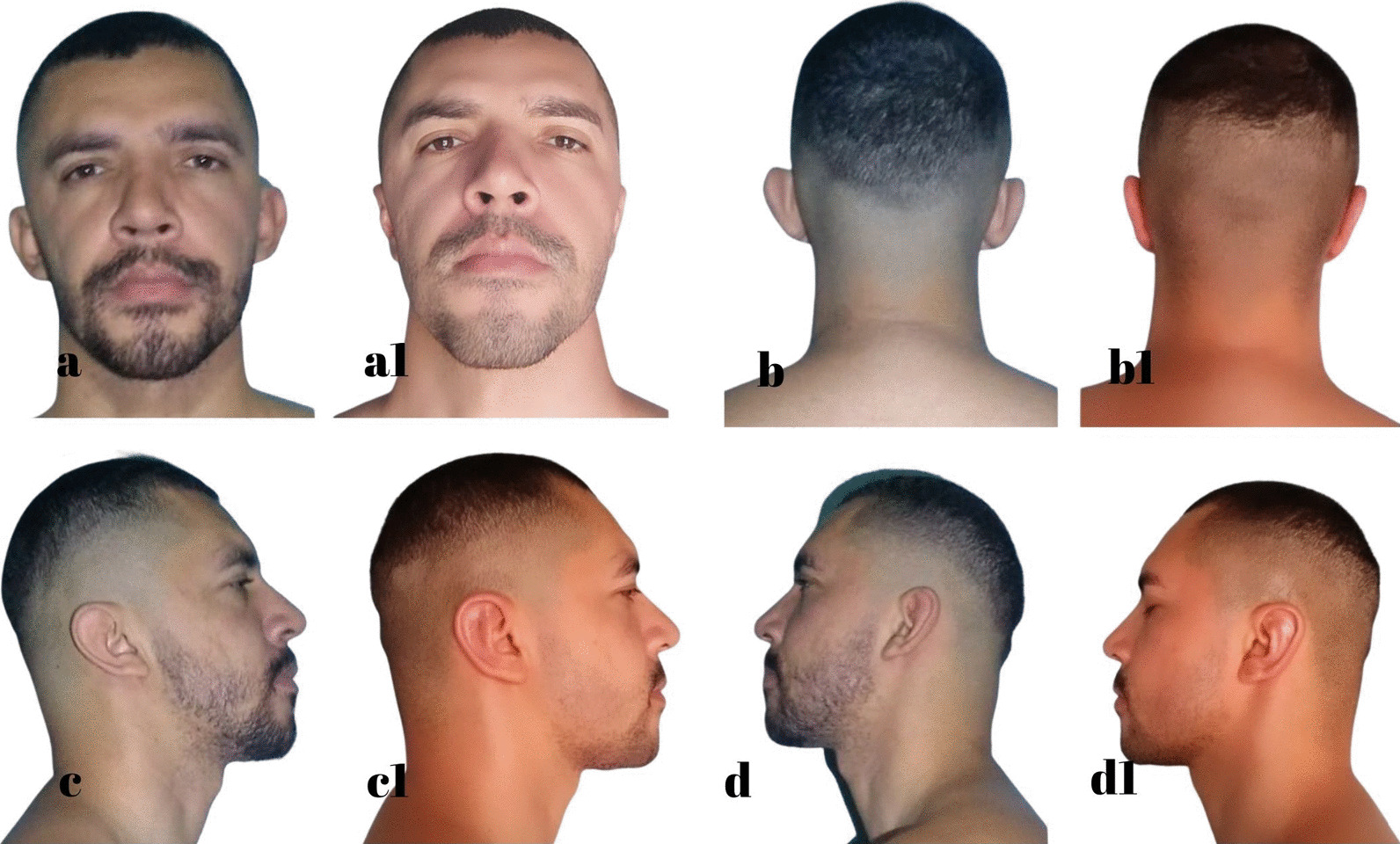
M.R.S. 38 years, **a**, **b**, **c**, **d** Before otoplasty. **a1**, **b1**, **c1**, **d1** 10 months after surgery. Source: Personal archive

**Fig. 12:**
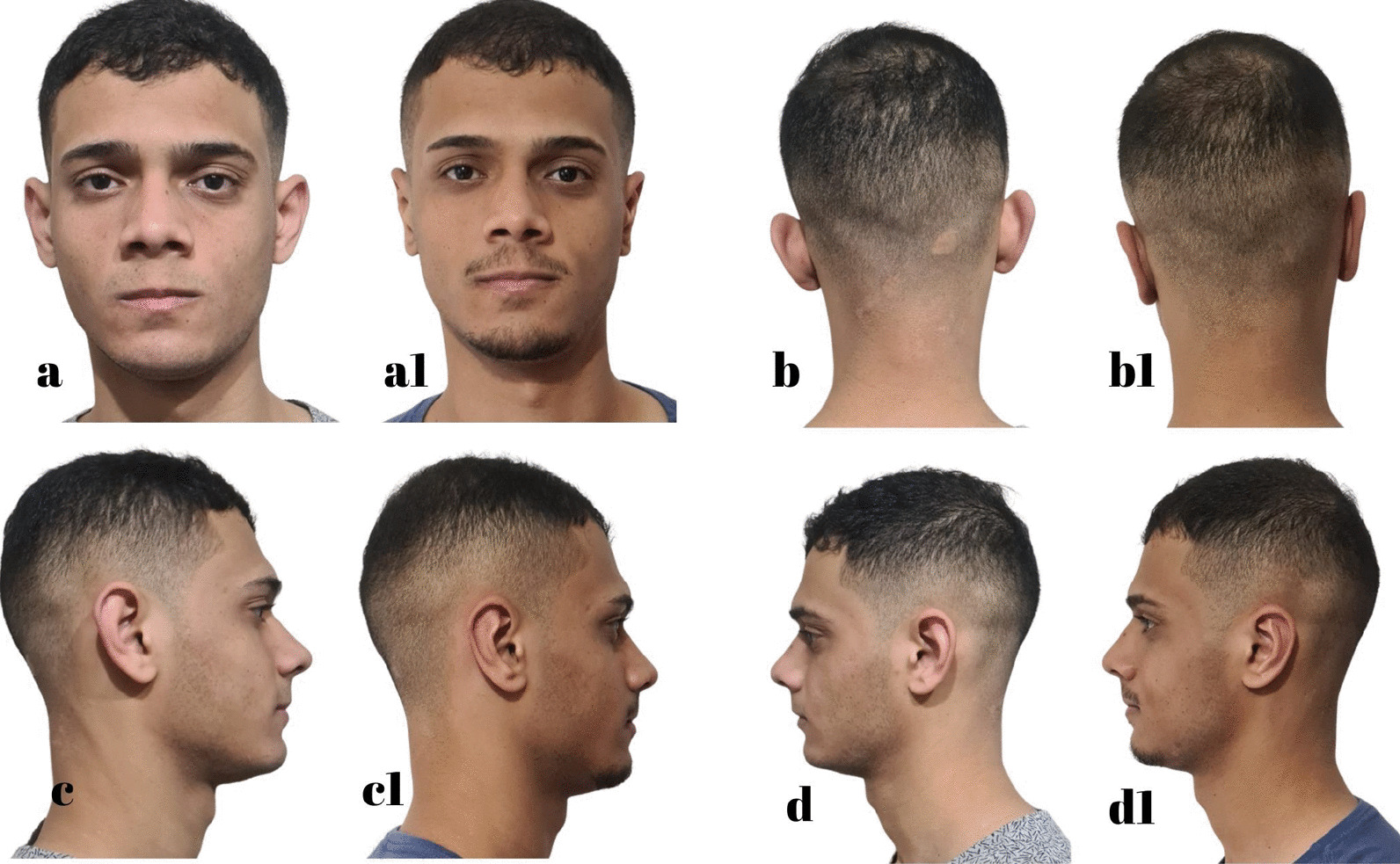
D.A.S. 21 years, **a**, **b**, **c**, **d** Before otoplasty. **a1**, **b1**, **c1**, **d1** 7 months after surgery. Source: Personal archive

The standard dressing was kept for 5 days, and then the patient was instructed to wear a ballet-style compression bandage only at night to sleep for a minimum period of 30 days. The following are some of the outcomes of this technique (Figs. 7, 8, 9, 10, 11, 12).

### Consent

Informed consent was obtained from all individual participants and parents/legal guardians in case of minor participants included in the study.

### Patient consent

Patients signed informed consent regarding publishing their data and photographs. The participant has consented to the submission of the case reports to the journal.

### Outcomes

The performance-optimized otoplasty technique proved to be efficient in reducing the surgical time to less than 45 min (total bilateral treatment time) compared to the average time of conventional techniques, which is approximately 95 min [12, 13]. It also allowed us to perform all surgeries under local anesthesia and sedation in an outpatient setting, with a hospital stay of approximately 2 h and 15 min. Furthermore, we did not use non-absorbable sutures in the cartilage, which in some cases can cause extrusions, infections, or chronic pain [9].

The new design of the antihelix cartilage resulted in a harmonious shape, without the appearance of operated ears in 98.6% of cases.

Table 1 shows the adherence criteria adopted in the study during the 1-year period.

To analyze the satisfaction of the 213 patients studied, we used the criteria based on objectives and satisfaction with results described by McDowell/Wright [15], as shown in Table 2.

**Table 1:** 1-Year adherence criteria for the patients studied

Characteristics	N = 213	Number (%)
Sex	Male	77 (36.15%)
	Female	136 (63.84%)
Mean age		21
Comorbidities	Large lobe	11 (5.16%)
Unilateral		0 (0%)
Bilateral		213 (100%)

**Table 2:** Surgical objectives in McDowell/Wright otoplasties

Number	Objectives		
1	Correction of upper third protrusion		
2	The helix of both ears should be in front of the anti-helix in a frontal view		
3	Harmonic and regular antihelix along the ears		
4	Aligned and undistorted retroauricular sulcus		
5	Retroauricular opening between 15 and 20 mm		
6	Symmetrical positioning of the ears		

Objectives	Good outcome	Average outcome	Bad outcome
Very satisfied (9–10)	160	30	0
Satisfied (7–8)	16		0
Somewhat satisfied (5–6)	0	4	0
Dissatisfied (< 5)	0	0	3

## Conclusion

The patients in this study demonstrated satisfaction with the results obtained by the otoplasty technique presented, with quick recovery and return to their daily activities, making this a viable alternative for performing surgeries in an outpatient setting, with low operating costs.

## Data Availability

Data sets are available by friendly request to the corresponding author.

## References

[1] Thorne CHMD, Wilkes GMD (2012). Ear deformities, otoplasty, and ear reconstruction. Plast Reconstr Surg..

[2] García-Purriños F, Raposo A, Guilllén A, Calero J, Giribet A, Barrios A (2019). Otoplasty using the combined Mustardé-Furnas technique: satisfaction and objective results. Aesthet Surg J.

[3] Boroditsky ML, Van Slyke AC, Arneja JS (2020). Outcomes and complications of the Mustardé otoplasty: a "good-fast-cheap" technique for the prominent ear deformity. Plast Reconstr Surg Glob Open.

[4] Lee Y, Kim YS, Lee WJ, Rha DK, Kim J (2018). Proposal of a classification system for the assessment and treatment of prominent ear deformity. Aesthet Plast Surg..

[5] Steven S, Kent HB (1999). Decoding the DNA of the Toyota production system. Harv Bus Rev.

[6] Furnas DW (1968). Correction of prominent ears by conchamastoid sutures. Plast Reconstr Surg.

[7] Peter A, Becky L, Guy J. Otoplasty: critical review of clinical results. The Laryngoscope. 10.1288/00005537-199108000-00013 1865738

[8] Smittenberg MN, Marsman M, Veeger NJGM, Moues CM (2018). Comparison of cartilage-scoring and cartilage-sparing otoplasty: a retrospective analysis of complications and aesthetic outcome of 1060 ears. Plast Reconstr Surg.

[9] Benoit IMM, Hendrickx MH, Assaf Z, Andrew G (2018). The ‘WiFi’ otoplasty: combined concentric posterior microchondrectomies and sutures for correction of prominent ears. J Plast Reconstr Aesthet Surg..

[10] Jones ES, Gibson JAG, Dobbs TD, Whitaker IS (2020). The psychological, social and educational impact of prominent ears: a systematic review. J Plast Reconstr Aesthet Surg..

[11] Gasques JA, Pereira de Godoy JM, Cruz EM (2008). Psychosocial effects of otoplasty in children with prominent ears. Aesthet Plast Surg..

[12] Nazarian R, Eshraghi AA (2011). Otoplasty for the protruded ear. Semin Plast Surg.

[13] Wright WK (1970). Otoplasty goals and principles. Arch Otolaryngol..

[14] Goulart FO, Arruda DSV, Karner BM, Gomes PL, Carreirão S. Correction of prominent ears by the cartilaginous incision technique, definition of the antihelix with Mustardé sutures and fixation of the cartilage at the mastoid. Rev Bras Cir Plast. 2011;26(4):602–7.

[15] McDowell AJ. Goals in otoplasty for protruding ears. Plast Reconstr Surg. 1968;41(1):17–27. 10.1097/00006534-196801000-00004. 10.1097/00006534-196801000-00004 5639206

